# The relationship of telomere length to baseline corticosterone levels in nestlings of an altricial passerine bird in natural populations

**DOI:** 10.1186/s12983-016-0133-5

**Published:** 2016-01-12

**Authors:** Verónica Quirici, Claudia Jimena Guerrero, Jesse S. Krause, John C. Wingfield, Rodrigo A. Vásquez

**Affiliations:** Departamento de Ecología y Biodiversidad, Facultad de Ecologíam y Recursos Naturales, Universidad Andres Bello, República 440, Santiago, Chile; Escuela de Medicina Veterinaria, Talca, Facultad de Recursos Naturales y Medicina Veterinaria, Universidad Santo Tomás, Av. Carlos Schorr 255, Talca, Maule Chile; Department of Neurobiology, Physiology and Behavior, University of California, One Shields Avenue, Davis, CA 95616 USA; Instituto de Ecología y Biodiversidad and Departamento de Ciencias Ecológicas, Facultad de Ciencias, Universidad de Chile, Las Palmeras 3425, Santiago, Chile

**Keywords:** Brood size, Latitudinal gradient, qPCR, Thorn-tailed rayadito

## Abstract

**Background:**

Environmental stressors increase the secretion of glucocorticoids that in turn can shorten telomeres via oxidative damage. Modification of telomere length, as a result of adversity faced early in life, can modify an individual’s phenotype. Studies in captivity have suggested a relationship between glucocorticoids and telomere length in developing individuals, however less is known about that relationship in natural populations.

**Methods:**

In order to evaluate the effect of early environmental stressors on telomere length in natural populations, we compared baseline corticosterone (CORT) levels and telomere length in nestlings of the same age. We collected blood samples for hormone assay and telomere determination from two geographically distinct populations of the Thorn-tailed Rayadito (*Aphrastura spinicauda*) that differed in brood size; nestlings body mass and primary productivity. Within each population we used path analysis to evaluate the relationship between brood size, body mass, baseline CORT and telomere length.

**Results:**

Within each distinct population, path coefficients showed a positive relationship between brood size and baseline CORT and a strong and negative correlation between baseline CORT and telomere length. In general, nestlings that presented higher baseline CORT levels tended to present shorter telomeres. When comparing populations it was the low latitude population that presented higher levels of baseline CORT and shorter telomere length.

**Conclusions:**

Taken together our results reveal the importance of the condition experienced early in life in affecting telomere length, and the relevance of integrative studies carried out in natural conditions.

## Background

Environmental stressors experienced by nestlings during development are of paramount importance because of potential effects on adult health and survival [1, 2]. In nestlings of altricial bird species, where the newly hatched young lack down and mobility, are not able to obtain food on their own, and must therefore be cared for by adults, environmental stressors are buffered by parents. However, even where parental quality is high, offspring exposure to a number of environmental and/or social perturbations like food shortages, parasites, pollutants, and sibling competition cannot be avoided [3–9].

Such perturbations activate the hypothalamic-pituitary-adrenal (HPA) axis, resulting in increased levels of the steroid hormone corticosterone (CORT), the primary glucocorticoid in birds [10, 11]. The HPA axis is often divided into two components: baseline glucocorticoids levels and stress response glucocorticoids levels [12]. The first is an approximation of the seasonal baseline level that the animal should maintain to be able to cope with the predictable demands of the current life-history stage [13, 14], so it reflects long-term adaptation. The former, (stress response: the increase in baseline glucocorticoids levels to the level reached in 30 min) best reflect short-term plastic responses to environmental perturbations [15]. Elevated levels of CORT during development can have adverse effects on growth, behaviour, metabolic rate, cognitive ability, and the immune system [16–22], with potentially negative consequences for the individual later in life [23]. Elevated levels of CORT can furthermore negatively affect the metabolic system of the organism via increased oxidative damage by reactive oxygen species (ROS), which are an unwanted product of metabolism [24, 25]. As the nucleobase guanine is a major oxidation target for ROS, the (TTAGGG)n repeats that constitute vertebrate telomeres are particularly vulnerable to oxidative attack [26].

Telomeres are chromosome caps, made of non-coding DNA repeats and associated proteins that protect the terminal ends of linear chromosomes (reviewed by [27]). They are essential to genome integrity because they protect chromosomes from degradation and help to ensure proper replication processes [28]. When telomeres reach a critical shortness, further cell division can damage coding DNA and so cells stop dividing and enter a state of replicative senescence or undergo programmed cell death [29, 30]. Consequently, telomere length appears to predict remaining lifespan in a variety of taxa [31–40].

It used to be thought that telomere loss (telomere shortening) occurred at a constant rate in individuals, and telomere length could therefore act as an internal ‘mitotic clock’ to measure the chronological age of organisms in the wild [41]. However, telomere lengths may vary between individuals of the same age due to variation in telomere inheritance [42, 43] but more importantly due to the rate by which telomeric base pairs are lost following oxidative stress [44, 45]. In addition, telomere loss occurs at a higher rate early in life when growth and development are still occurring [46–48]. Thus, environmental conditions early in life are an important factor in determining telomere lengths in adulthood [49] and subsequently survival (e.g. [37]).

For instance, Haussman and collaborators [50] observed that chicks of the domestic chicken (*Gallus domesticus*) exposed to experimentally increased CORT levels (via CORT injected eggs) had a higher frequency of shorter telomeres compared with control birds and low CORT treatment, at the age of 21 days old. Recently, Tissier and collaborators [51] chronically treated pre-laying zebra finch females (*Taeniopygia guttata*) with CORT (via CORT injections) and observed that CORT treatment decreased growth rate in male chicks and increased rate of telomere loss in mothers and female offspring.

These studies have helped in the understanding of the complex relationship between stress (CORT and oxidative stress) and telomere length in individuals in captivity during their development. In order to evaluate if this relationship exists in natural conditions, we analysed baseline CORT and telomere length in nestlings of the same age in the altricial passerine, the Thorn-tailed Rayadito (*Aphrastura spinicauda).*

The Thorn-tailed Rayadito *Aphrastura spinicauda* (Furnariidae: Passeriformes) is an insectivorous and endemic species of the South American temperate forest [52]. The Thorn-tailed Rayadito has a socially monogamous mating system where both members of the pair contribute to nest building, incubation, and feeding of nestlings [53]. They are small (11 g) and lay one clutch per breeding season during the austral spring [54]. Eggs are laid on alternate days and the Thorn-tailed Rayadito postpones incubation until after the clutch is complete. The nest construction period lasts 6–15 days, the incubation period lasts 9–15 days and fleding occur at 20–21 days old. On day 13 nestling attained asymptotic size with respect to tarsus, bill length and body mass, but not with respect to wing length [54]. Clutch size varies latitudinally with a mean of 2.5 eggs observed in the low latitude population (North, 30 °S), and a mean of 5.5 eggs in the high latitude population (South, 55 °S) [55] (Table 1), a life-history trait that can influence baseline CORT levels (at bigger brood size, higher baseline CORT level [56, 57]). In addition, nestling of the high latitude population presents higher body mass, a condition that can influence baseline CORT (at lower body mass, higher baseline CORT level [58]) (Table 1). Finally, the low latitude population presents lower annual temperatures, higher annual precipitations and higher productivity than the high latitude population (Table 1). Because of these differences in ecological characteristics, brood size and nestling body mass, we investigate (i) whether the magnitude of the relationship between baseline CORT and telomere length vary in each population in relation to brood size and nestling body mass (path analysis); and (ii) whether nestling of the two population differed in baseline CORT levels and telomere length.

**Table 1:** Geographical origin, climatic data, values of reproductive function (e.g. clutch size) and biometry (mass of nestlings) for two populations of Rayaditos during the study year 2012

Population	Precipitations (SD)	Coefficient of variation	Temperature (SD)	Coefficient of variation	DMi (SD)	Coefficient of variation	Clutch size	Nestling mass
North	124.63	1.23	11.18	17.31	0.45 (0.34)	120.64	2.44 (0.64)	12.75 (1.88)
(33° S)	(1.83)		(1.89)					
South	318.35	0.57	4.35	9.45	2.10 (1.13)	12.00	4.69 (0.89)	14.66 (1.25)
(55° S)	(1.50)		(4.12)					

The de Martonne (DMi) aridity index was calculated following di Castri and Hajek (1976), based on the average of monthly temperatures and precipitation. The index is low in hot, dry deserts (low productivity) and high in cool, wet areas (high productivity). The mean annual temperature (calculated as the average of the monthly temperatures) and total annual precipitation are reported. The variances of precipitation (σ^2^ P) temperature (σ^2^ T) and de Martonne index (σ^2^ DMi) were estimated from the mean of monthly values

*DMi* Martonne aridity index, *Clutch size* number of eggs, *Nestling mass (g)* weight of nestlings at 13 days old

Climatic data were collected from < http://www.ceazamet.cl, http://www.meteochile.gob.cl/

## Methods

### The study population

**Fig. 1:**
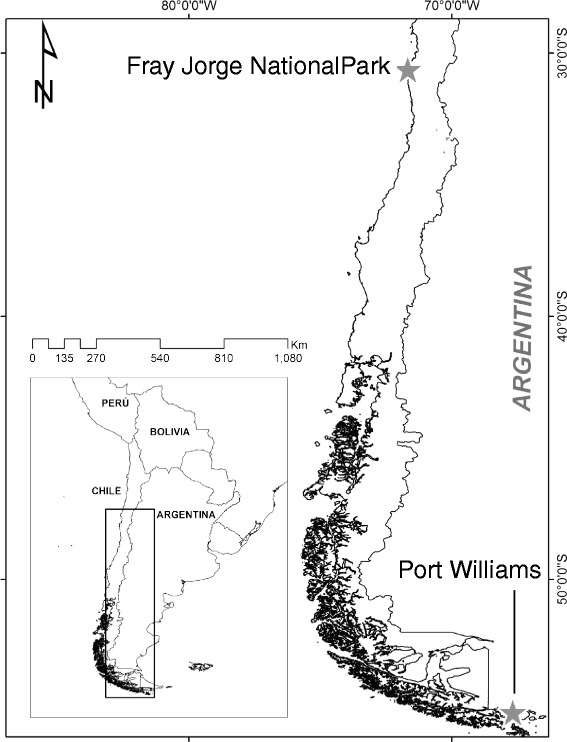
Location of the two Rayadito populations. Low latitude population: Fray Jorge National Park (30°38’ S, 71°40’ W); high latitude population: Port Williams, Navarino Island (55°40’ S, 67°40’ W) in Chile

Because Thorn-tailed Rayaditos are secondary cavity nesters [52] they easily adopt to nesting in artificial boxes. As part of a long-term study, we have monitored nest boxes in different populations throughout Chile [55]. In the present study we compared baseline CORT levels and telomere length in the two populations at the extremes of the geographic distribution (Fig. 1). The low latitude population in our experiment, and the northern limit of the species distribution, is located in Fray Jorge National Park (30°38’ S, 71°40’ W), which is a relic forest composed mainly of Olivillo (*Aextoxicon punctatum*), occurring in patches at the top of the coastal mountain range, where fog-induced microclimatic conditions allow the forest to exist in this semiarid region. Our high latitude population, is located in Navarino Island (55°4’ S, 67°40’ W). At this site, the vegetation is characterized by deciduous Magellanic forest, whose characteristic species are Lenga Beech (*Nothofagus pumilio*), and Ñirre Beech (*Nothofagus antarctica*).

### Monitoring, capture procedures and blood sampling

Each year nest boxes were monitored on a weekly basis until they were occupied, at which point the frequency of monitoring was increased to detect lay (date of first egg) and hatch dates (hatching synchrony). All nestlings were removed from the nest at 12–14 days post-hatch to have blood samples between 7:30 am and 12:30 pm. Nestlings were removed individually, carried away from the nest (20–40 m) and a small blood sample (approximately 50 μL) was obtained by puncturing the brachial vein with a sterile needle and extracting blood into heparinized micro-hematocrit capillary tubes and banded with individual metal bands (National Band and Tag Co., Newport, Kentucky, USA, or Split Metal Bird Rings, Porzana Ltd, UK or with a numbered band provided by the Servicio Agrícola y Ganadero (SAG), Chile). Each nestling was located in a clutch bag and a subsequent nestling was removed and similarly processed. In order to minimize blood sampling time, we weighted nestling after all nestlings were blended. Samples were stored on ice until the end of the sampling period (maximum of 5 h) and were then centrifuged for 5 min at 8000 rpm to separate the plasma from the red blood cells. The plasma was aspirated with a Hamilton syringe and stored (at − 20 °C) until assayed for total CORT content (University of California, Davis). The red blood cells were stored in FTA Classic Cards (Whatman®) for subsequent molecular sex determination. As CORT levels are known to rise after 3 min in adults, we performed Pearson correlation between the time of sampling relative to the initial nest disturbance (first chick removed) and baseline CORT on each nestling within each nest box. CORT levels did not increase with sampling times in the low latitude population (*r* = 0.032, *P* = 0.88), which ranged from 45 to 1320 s and in the high latitude population (*r* = 0.16, *P* = 0.56), which ranged from 50 to 1500. In addition, because baseline CORT levels can drop between the periods we took blood samplings (7:30 to 12:30), we correlated the time at which we took blood samplings of a nest box with the mean baseline CORT levels to each nest box. Mean baseline CORT levels did not increase with sampling times in the low latitude population (*r* = 0.024, *P* = 0.94) or in the high latitude population (*r* = 0.14, *P* = 0.66). A total of 93 blood samples were collected, 33 from the low latitude population (12 nest boxes) and 60 from the high latitude population (13 nest boxes). This study was carried out with the permission of SAG and the Corporación Nacional Forestal (CONAF), Chile.

### Molecular sexing

Because Thorn-tailed Rayadito nestlings present an absence of sexual dimorphism we used a previously described molecular sexing method [55]. Briefly, DNA was extracted using a commercial kit (QIAGEN Inc., Valencia, CA). The sex of nestlings was determined using 2550 F and 2718R primers [59]. Polymerase Chain Reaction (PCR) products were run in 1 % agarose gels, pre-stained with ethidium-bromide, and detected in a Fluorimager (Vilber Lourmat). Birds were sexed as females (heterogametic: WZ) when the CHD1W of 450 bp and CHD1Z of 600 bp fragments were amplified, and identified as males (homogametic: ZZ) when only the CHD1Z of 600 bp fragments were present.

### Hormone assay

Plasma concentrations of CORT were determined using a direct radioimmunoassay. To determine the efficiency of steroid extraction from the plasma, 2000 cpm of tritiated CORT was added to all samples and incubated overnight. Steroids were extracted for 3 h from the plasma using freshly re-distilled dichloromethane. The aspirated dichloromlethane was dried using a stream of nitrogen at 35 °C. Samples were reconstituted in phosphate-buffered saline with gelatin. All samples were run in duplicate, intra-assay variation for CORT was 9.20 %, inter-assay variation was 9.28 %.

### Telomere determination

Telomeres were determined using a real-time quantitative PCR technique validated for birds [60, 61]. After extracting genomic DNA (as described in ‘Molecular sexing’ above), we quantified the purity of the DNA using a Nanodrop 1000 spectrophotometer (Thermo Scientific). We followed the protocol described by Angelier and collaborators [39]. A control single-copy gene (glyceraldehyde-3-phosphate dehydrogenase, GAPDH) was amplified using the primers GAPDH-F (5’-TGACCACTGTCCATGCCATCAC-3’) and GAPDH-R (5’TCCAGACGGCAGGTCAGGTC-3’). Telomere primers were Tel1b (5’-CGGTTTGTTTGGGTTTGGGTTTGGGTTTGGGTTTGGGTT-3’) and Tel2b (5’-GGCTTGCCTTACCCTTACCCTTACCCTTACCCTTACCCT-3. qPCR for both telomeres and GAPDH was performed using 4 ng per reaction. Both the telomere primers (Tel1b and Tel2b) and the GAPDH primers (GAPDH_F and GAPDH_R) were used at a concentration of 400 nM (Tel1b and GAPDH_F) and 900 nM (Tel2b and GAPDH_R), in a final volume of 20 ul containing 10 ul of Brillant II SYBER Green qPCR Master Mix (Stratagene, La Jolla, CA, USA). Then the forward primer (GAPDH_F or Tel1b), the reverse primer (GAPDH_R or Tel2b) and a reference dye was added to the 2x Brilliant II SYBR Green master mix in this specific order. After gently mixing the reaction, the Rayadito DNA was added and the reaction mixed. Telomere and GAPDH real-time amplifications were performed in two different plates using a Step-One™ Real-Time PCR System (Life Technologies, USA). We used PCR conditions as follow: 20 s at 95 °C followed by 30 cycles of 3 s to 95 °C, 10 s to 54 °C and 20 s at 60 °C. GAPDH PCR conditions were 20 s at 95 °C followed by 40 cycles of 3 s to 95 °C, 10 s to 62 °C and 20 s at 60 °C.

To test the efficiency of each PCR reaction (accepted efficiency range 100 ± 15 %), a standard curve was produced in every 48 well plate by serially diluting a pool of Rayadito DNA (80, 40, 20, 10, and 5 ng ml ^−1^) and by running it in triplicate. To be able to compare measurements among plates, one individual was used as a reference and run in triplicate on every plate. The threshold C_t_ of this reference sample was then calculated for each plate; the C_t_ of a DNA sample is the fractional number of PCR cycles to which the sample must be subjected in order to accumulate enough products to cross a set threshold of magnitude of fluorescent signal. All other samples were run in duplicate on the plates, and mean values per plate were used to calculate relative T/S ratios for the target individual relative to the reference individual using the formula 2^ΔΔCt^, en where ΔΔC_t_ = (C_t_ Tel - C_t_ GAPDH)_reference_ - (C_t_ Tel - C_t_ GAPDH)_target_ . Quantitative PCR was performed a minimum of two times (i.e. two telomere and two GAPDH plates) for each sample, and mean T/S ratios were used in the statistical analyses. The mean intraplate coefficient of variation was 3.32 per cent for the C_t_ values of GAPDH assays and 4.79 per cent for the C_t_ values of telomere assays.

### Statistical analyses

To examine the relationship between telomere length and CORT levels, we correlated these variables performing a path analysis [62]. Because baseline CORT can be affected by brood size [56, 57] and body mass [58] we included these variables in the analysis. In order to avoid pseudoreplication (sibling belong to the same nest box) we calculated mean values of baseline CORT and telomere length for each nest box and used these values in the path analysis.

We examined the effects of population (two levels – fixed effect) and sex (two levels – fixed effect) on baseline CORT (ng/ml) and on telomere length using linear mixed effect models (LMM). Variance components were estimated using restricted maximum likelihood. Brood size was included as a random factor and the nest was included as a random factor to control for siblings (pseudoreplication). Because baseline CORT level could be correlated with body mass (lower body mass, higher baseline CORT level, refer 58], we included body mass as a covariate. We chose weight instead of the residuals between tarsus length and body weight [63], because it has been proposed as a better predictor of body condition [64]. CORT levels, body mass and telomere length were logarithmically transformed prior to analysis to fit assumptions of normality for parametric tests. All statistical tests were two-tailed and conducted using IBM SPSS Statistics 20. Data are reported as mean ± SD.

## Results

**Fig. 2:**
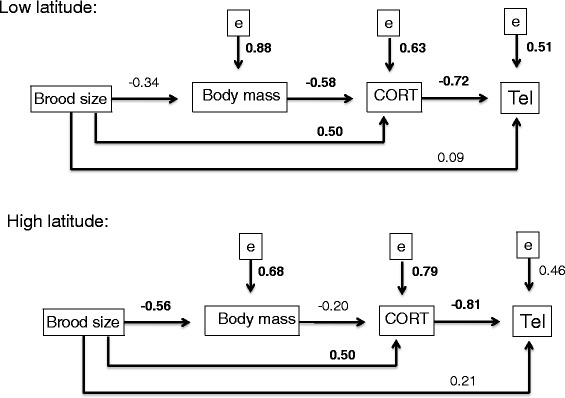
Path analysis of the relationship between clutch size, baseline CORT and telomere length in the low latitude and high latitude populations. Path analysis was conducted with average values of nest boxes (low latitude, *N* = 12 nests; high latitude, *N* = 13 nests). Also shown are the residuals (e1, e2), which combine all unexplained effects and measurement errors. Indicated are the path coefficients (*cp*). The path coefficients give the strengths of the relationships between pairs of variables when the influences of previous variables are accounted for. Values in bold indicate path coefficients statistically significant (*P* < 0.05)

**Fig. 3:**
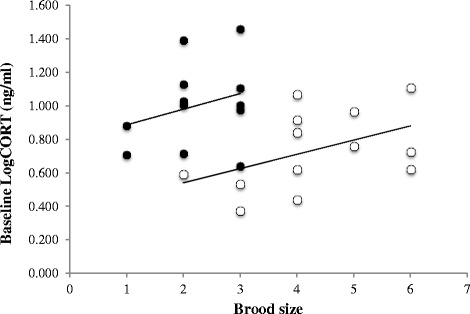
Association between brood size and baseline CORT. Correlation between brood size and mean Log baseline CORT (ng/ml) per nest box (low latitude, *N* = 12; high latitude, *N* = 13) in the low latitude population (*black circles*) and the high latitude population (*white circles*). Correlation values are reported in Fig. 4

**Fig. 4:**
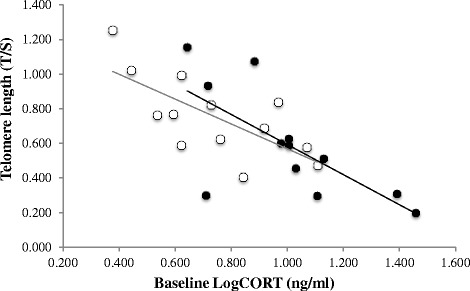
Association between baseline CORT and telomere length. Correlation between mean Log baseline CORT (ng/ml) and mean Log telomere length (T/S ratio) per nest box (low latitude, *N* = 12; high latitude, *N* = 13) in the low latitude population (*black circles*) and the high latitude population (*white circles*). Correlation values are reported in Fig. 4

We tested the hypothesis that brood size affects baseline CORT and how this affects telomere length. In addition we tested the hypothesis that brood size affects body mass and how this affects baseline CORT. Finally we tested the hypothesis that brood size affects telomere length. In the low latitude population, brood size had a negative effect on body mass (*pc* = −0.34) and body mass had a negative effect on baseline CORT (*pc* = −0.58) (Fig. 2). Brood size had a positive effect on baseline CORT (*pc* = 0.50) (Figs. 2 and 3). The path coefficient between baseline CORT and telomere length had a strong and negative effect (*pc* = −0.72) (Figs. 2 and 4) and represents the effect of baseline CORT on telomere length for a given brood size, not the overall effect of baseline CORT on telomere length.. In the high latitude population brood size had a negative effect on body mass (*pc* = −0.56) and body mass had a negative effect on baseline CORT (*pc* = −0.20) (Fig. 2). Brood size had a strong and positive effect on baseline CORT (*pc* = 0.50) (Figs. 2 and 3). The path coefficient between baseline CORT and telomere length had a strong and negative effect (*pc* = −0.81) (Figs. 2 and 4).

**Fig. 5:**
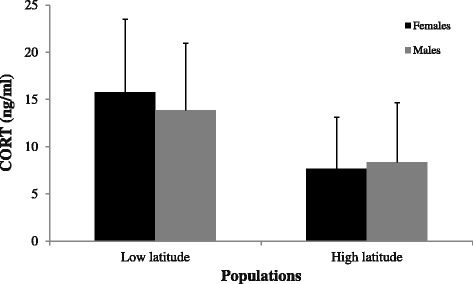
Baseline levels of nestlings. Average baseline CORT levels (ng/ml ± SD) of 12 days old nestlings in the low latitude (Fray Jorge National Park) and in the high latitude population (Navarino Island) of males (*gray*) and females (*black*). Low latitude population, *N* = 16 males and 17 females. High latitude population, *N* = 35 males and 25 females

Rayaditos from the low latitude population presented higher levels of baseline CORT than their high latitude counterparts (F_1,12.99_ = 4.76, *p* = 0.05) (Fig. 5). There was no effect for sex alone (F_1,74.69_ = 0.04, *p* = 0.85), effect from body mass (F_1,59.27_ = 1.71, *p* = 0.19), or an interaction between population and sex (F_1,76.81_ = 2.07, *p* = 0.15).

**Fig. 6:**
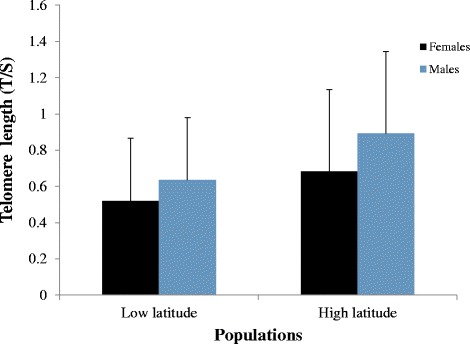
Telomere length of nestlings. Average telomere length (T/S ratio ± SD) of 12 days old nestlings in the low latitude (Fray Jorge National Park) and in the high latitude population (Port Williams, Navarino Island) of males (*gray*) and females *(black*). Low latitude population, *N* = 16 males and 17 females. High latitude population, *N* = 35 males and 25 females

Telomere length of nestlings of the same age tends to be shorter in the low latitude population (F_1,18.74_ = 3.69, p = 0.07) (Fig. 6). There was no effect of sex (F_1,76.72_ = 0.74, *p* = 0.39), effect from body mass (F_1,67.70_ = 0.04, *p* = 0.85) or an interaction between population and sex (F_1,77.03_ = 0.15, *p* = 0.69).

## Discussion

The objective of our study was to evaluate the relationship between baseline CORT and telomere length in nestlings of the Thorn-tailed Rayadito of the same age from two populations that differed in ecological and life history characteristics. Within population we observed a direct and negative relationship between baseline CORT levels and telomere length (see bellow); between populations we observed higher baseline CORT levels and a trend toward shorter telomere length in the high latitude population than in the low latitude population.

### Baseline CORT levels and telomere length

The elevation of baseline plasma levels of CORT presents a potential negative consequence for the organism, through an increase in oxidative damage [24, 25] and ultimately shortening telomeres [26]. We observed a trend for shorter telomeres in the low latitude population (*p* = 0.07), the population that presented higher baseline CORT levels (*p* = 0.05). In addition, we found a statistically negative significant relationship between baseline CORT and telomere length (path coefficients, *p* < 0.05) in both populations. Taking these results together (shorter telomeres in the low latitude population and a negative relationship between CORT levels and telomere length), we suggest that baseline CORT negatively affects telomere length by shortening them, presumably via oxidative stress. For instance, recently Stier and collaborators [65] observed that 10 days old chicks of the king penguin (*Aptenodytes patagonicus*) that born late in the season had higher CORT and oxidative stress levels and shorter telomere length than those chicks that born early in the seasons. Although the results of the aforementioned study and the result of our study in wild populations, together with experimental studies [50, 51, 66], goes in the same direction and suggest a strong association between stress (baseline CORT and/or oxidative stress) and telomere length, we cannot discard other mechanism that could affect telomere length, like alterations of early growth trajectories (e.g. [67]). Anyway the results of these investigations are interesting because it may help us to understand early life factors that determine telomere length, in particular considering that telomere attrition is higher during early life when growth and development are still occurring [46–48] and longitudinal analysis has demonstrated that telomere length in developing individuals, rather than in adulthood, is a strong predictor of lifespan, at least in *Taeniopygia guttata*- [37]. In a concurrent study, we are carrying out a Capture-Mark-Recapture study (5 years of data) in order to evaluate apparent survival probabilities in relation to telomere length when nestlings were 12 days old in both populations. If early telomere length were a strong predictor of lifespan, we would predict a lower survival probability in the low latitude population.

In addition, in order to evaluate the role of CORT on telomere dynamics it would be interesting to investigate the rate of telomere loss of nestlings during developing (for an example in captivity, [51]) in both population, to evaluate if the magnitude of telomere loss is higher in the low latitude population, the population that presents higher levels of baseline CORT. Changes in the magnitude of telomere loss in relation to the environment have been registered in adult’s birds. Angelier and colaborators [39] showed that telomeres of individuals of the American redstart (*Setophaga ruticilla*) that wintering in a low-quality habitat shorten more than those of individuals wintering in a high-quality habitat and in black-legged kittiwakes (*Rissa tridactyla*) those individuals that have been experimentally stressed during the reproductive season, presented accelerated telomere loss when recaptured at the next year [67].

Finally, the results of our path analysis suggest that telomere length decreased with an increase in baseline CORT levels and baseline CORT increased with brood size. Increasing the number of nest boxes sampled would have improved experimental validity, however path coefficients were still statistically significant. This result suggests that a competitive environment when the brood is larger (due to sibling competition) increased baseline CORT resulting in shorter telomere length. Others researchers have investigated the effect of early adversity on telomeres in altricial birds by manipulating brood size [40, 68], showing that telomere attrition accelerates in chicks in large broods, having a negative effect on survival at the next year [40]. However in our study brood size alone did not have a significant effect on telomere length in any of the two populations (low latitude: *cp* = 0.09, high latitude: *cp* = 0.21). Our result is in concordance with the recent experiments of Nettle and collaborators [69] in European starlings (*Sturnus vulgaris*), they demonstrated that rather than brood size per se, what accelerated telomere attrition was early life competitive disadvantage (for the smaller birds amongst the siblings in the nest). Our second result also is in agreement with the aforementioned study, although the magnitudes of the associations vary in both populations, we observed that baseline CORT decreased with an increase in body mass and body mass decrease with an increased in brood size ((in the low latitude population, we observed weaker association between brood size and body mass (*pc* = −0.34) than between body weight and CORT (*pc* = −0.58), while in the high latitude population we observed stronger association between brood size and body weight (pc = −0.56) than between body mass and CORT (*pc* = −0.20)). Summarizing, although we did not manipulate brood size, within each population we observed higher baseline CORT levels in nestlings of bigger brood sizes (*pc* = 0.50 in both populations) and shorter telomeres in those nestlings.

### Differences in baseline CORT levels of nestlings between populations

We observed that nestlings of Thorn-tailed Rayadito presented higher baseline CORT levels in the low latitude population than their high latitude counterparts. This pattern replicates the baseline CORT levels of Thorn-tailed Rayadito’s adults [55].

So, why did nestlings have the same pattern of baseline CORT as adults? One possible scenario is that the pattern we observed was the result of maternal effects either via maternally derived glucocorticoid deposition into the eggs [70, 71] or via parental care behaviour, such as provisioning rate and food quality [8, 72, 73]. On the other hand, given that these two populations are genetically distinct [74, 75], it is possible that differences exist in the set point of baseline CORT in individuals in each population (e.g., [76]), and hence a relatively higher CORT level in the low latitude population may not mean these individuals are “more stressed”.

Beyond the mechanism involved in determining baseline CORT levels and its possible behavioural consequences for nestlings such as increased begging [77, 78], increased sibling aggression [17], increased learning abilities [57] or in the preparation of nestlings to an stressor environment via organization of the H-P-A axis [49, 79, 80] what we can conclude from our study is that baseline CORT levels of nestlings follow the same pattern of baseline CORT levels of adults, that is to say higher levels in the low latitude population. The habitat of the low latitude population has been characterized as a isolated relic forest from the Pleistocene period, where fog-induced microclimatic conditions in the present day allow the forest to exist in an otherwise semiarid region, characterized by low primary productivity ([55], this study). So, one possibility is that baseline CORT levels of nestlings reflect habitat quality, as has occurred in adults of some species (721, [81–86]). However, we know little about if levels of baseline CORT reflect habitat quality in nestlings. To the best of our knowledge the only study that has evaluated levels of baseline CORT in nestlings inhabiting contrasting environments was the study of Blas and collaborators [87] in white storks (*Ciconia ciconia*). These authors observed that nestlings inhabiting crop fields had two-fold higher levels of baseline CORT when compared to nestlings inhabiting a marsh environment. One possibility is that the baseline CORT levels of Thorn-tailed Rayadito adults [55] and nestlings (the subject of this investigation) reflect habitat quality. For instance, low primary productivity and highly fragmented forest decrease the abundance of insects [88, 89], which forms the basis of the Thorn-tailed Rayadito”s diet. The sensitivity of Thorn- tailed Rayadito to differences in productivity should be not surprising. In general, habitat specialist species are particularly vulnerable to the negative impacts of poor quality habitat [90] and in particular, forest specialist bird species are particularly sensitive, as they only inhabit a single habitat type [88, 91].

## Conclusions

Within each population we propose brood size, via its effect on body mass, as the main determinant in the values of baseline CORT we observed and subsequently in affecting telomere length. In addition we observed that nestlings of the same age presented differences in baseline CORT between the high and low latitude populations. We cannot determine the mechanism of such differences, but we observed that nestlings follow the same pattern as adults. Whether this trend is the result of genetic differences or maternal effect should be considered in future investigations. Taken together our results indicate the importance of the environmental conditions experienced early in life in affecting telomere length, and the relevance of integrative studies carried out in natural conditions.

## References

[1] Lindström J (1999). Early development and fitness in birds and mammals. Trends Ecol Evol.

[2] Monaghan P (2008). Early growth conditions, phenotypic development and environmental change. Phil Trans R Soc B.

[3] Eeva T, Lehikoinen E (1996). Growth and mortality of nestling great tits (*Parus major*) and pied flycatchers (*Ficedula hypoleuca*) in a heavy metal pollution gradient. Oecologia.

[4] Love OP, Bird DM, Shutt LJ (2003). Plasma corticosterone in American kestrel siblings: effects of age, hatching order, and hatching asynchrony. Horm Behav.

[5] Neuenschwander S, Brinkhof MWG, Kölliker M, Richner H (2003). Brood size, sibling competition, and the cost of begging in great tits (*Parus major*). Behav Ecol.

[6] Osorno JL, Kuchar A, Möstl E, De la Martinez-Puente J, Lobato E, Merino S (2008). Corticosterone metabolites in blue tit and pied flycatcher droppings: Effects of brood size, ectoparasites and temperature. Horm Behav.

[7] Bize P, Stocker A, Jenni-Eiermann S, Gasparini J, Roulin A (2010). Sudden weather deterioration but not brood size affects baseline corticosterone levels in nestling Alpine swifts. Horm Behav.

[8] Rensel MA, Wilcoxen TE, Schoech SJ (2010). The influence of nest attendance and provisioning on nestling stress physiology in the Florida scrubjay. Horm Behav.

[9] Lynn SE, Kern MD (2013). Environmentally relevant bouts of cooling stimulate corticosterone secretion in free-living eastern bluebird (*sialia sialis*) nestlings: potential links between maternal behavior and corticosterone exposure in offspring. Gen Comp Endocrinol.

[10] Wingfield JC (1994). Modulation of the adrenocortical response to stress in birds.

[11] Sapolsky RM, Romero LM, Munck AU (2000). How do glucocorticoids influence stress-responses? Integrating permissive, suppressive, stimulatory, and adaptive actions. Endocr Rev.

[12] Romero LM (2004). Physiological stress in ecology: lessons from biomedical research. Trends Ecol Evol.

[13] Landys MM, Ramenofsky M, Wingfield JC (2006). Actions of glucocorticoids at a seasonal baseline as compared to stress-related levels in the regulation of periodic life processes. Gen Comp Endocrinol.

[14] Bókony V, Lendvai AZ, Liker A, Angelier F, Wingfield JC, Chastel O (2009). Stress response and the value of reproduction: are birds prudent parents?. Am Nat.

[15] Romero LM (2002). Seasonal changes in plasma glucocorticoid concentrations in free-living vertebrates. Gen Comp Endocrinol.

[16] Kuhn ER, Geris KL, van der Geyten S, Mol KA, Darras VM (1990). Inhibition and activation of the thyroidal axis by the adrenal axis in vertebrates. Comp Biochem Physiol A.

[17] Kitaysky AS, Kitaiskaia EV, Piatt JF, Wingfield JC (2003). Benefits and costs of increased levels of corticosterone in seabird chicks. Horm Behav.

[18] Rubolini D, Romano M, Boncoraglio G, Ferrari RP, Martinelli R, Galeotti P (2005). Effects of elevated egg corticosterone levels on be- havior, growth, and immunity of yellow-legged gull (*Larus michahellis*) chicks. Horm Behav.

[19] Saino N, Suffritti C, Martinelli R, Rubolini D, Møller AP (2003). Immune response co-varies with corticosterone plasma levels under experimentally stressful conditions in nestling barn swallows (*Hirundo rustica*). Behav Ecol.

[20] Spencer KA, Verhulst S (2007). Delayed behavioral effects of postnatal exposure to corticosterone in the zebra finch (*Taeniopygia guttata*). Horm Behav.

[21] Spencer KA, Verhulst S (2008). Post-natal exposure to corticosterone affects standard metabolic rate in the zebra finch (*Taeniopygia guttata*). Gen Comp Endocrinol.

[22] Müller C, Jenni-Eiermann S, Jenni L (2009). Effects of a short period of elevated circulating corticosterone on postnatal growth in free-living Eurasian kestrels *Falco tinnunculus*. J Exp Biol.

[23] Blas J, Bortolotti GR, Tella JL, Baos R, Marchant TA (2007). Stress response during development predicts fitness in a wild, long-lived vertebrate. Proc Natl Acad Sci U S A.

[24] Agostinho P, Cunha RA, Oliveira C (2010). Neuroinflammation, oxidative stress and the pathogenesis of Alzheimer’s disease. Curr Pharm Des.

[25] Constantini D, Marasco V, Møller AP (2011). A meta-analysis of glucocorticoids as modulators of oxidative stress in vertebrates. J Comp Physiol B.

[26] Bohr CT, Zhou F, Vallabhaneni H, Wang Z, Rhee DB, Lu J (2010). Characterization of oxidative guanine damage and repair in mammalian telomeres. PLoS Genetics.

[27] Xin H, Liu D, Songyang Z (2008). The telosome/shelterin complex and its functions. Genome Biol.

[28] Aubert G, Lansdorp PM (2008). Telomeres and aging. Physiol Review.

[29] Olovnikov AM (1996). Telomeres, telomerase, and aging: origin of the theory. Exp Gerontol.

[30] Campisi J (2003). Cellular senescence and apoptosis: how cellular responses might influence aging phenotypes. Exp Gerontol.

[31] Cawthon RM, Smith KR, O’Brien E, Sivatchenko A, Kerber RA (2003). Association between telomere length in blood and mortality in people aged 60 years or older. Lancet.

[32] Haussmann MF, Winkler DW, Vleck CM (2005). Longer telomeres associated with higher survival in birds. Biol Lett.

[33] Pauliny A, Wagner RH, Augustin J, Szep T, Blomqvist D (2006). Age-independent telomere length predicts fitness in two bird species. Mol Ecol.

[34] Bize P, Criscuolo F, Metcalfe NB, Nasir L, Monaghan P (2009). Telomere dynamics rather than age predict life expectancy in the wild. Proc Roy Soc Lond B.

[35] Salomons HM, Mulder GA, Van de Zande L, Haussmann MF, Linskens MHK, Verhulst S (2009). Telomere shortening and survival in free-living corvids. Proc Roy Soc Lond B.

[36] Foote CG, Daunt F, González-Solís J, Nasir L, Phillips RA, Monaghana P (2011). Individual state and survival prospects: age, sex, and telomere length in a long-lived seabird. Behav Ecol.

[37] Heidinger BJ, Blount JD, Bonera W, Griffiths K, Metcalfe NB, Monaghan P (2012). Telomere length in early life predicts lifespan. Proc Natl Acad Sci U S A.

[38] Barrett ELB, Burke TA, Hammers M, Komdeur J, Richardson DS (2013). Telomere length and dynamics predict mortality in a wild longitudinal study. Mol Ecol.

[39] Angelier F, Vleck CM, Holberton RL, Marra PP (2013). Telomere length, non-breeding habitat and return rate in male American redstarts. Func Ecol.

[40] Boonekamp JJ, Mulder GA, Salomons HM, Dijkstra C, Verhulst S (2014). Nestling telomere shortening, but not telomere length, reflects developmental stress and predicts survival in wild birds. Proc Roy Soc Lond B.

[41] Haussmann MF, Vleck CM (2002). Telomere length provides a new technique for aging animals. Oecologia.

[42] Bayer J, Thomas G, Kolvraa S, Graakjaer J, Der-Sarkissian H, Schmitz A (2006). Allele specific relative telomere lengths and inheritance. Human Genetics.

[43] Reichert S, Rojas ER, Zahn S, Robin J-P, Criscuolo F, Massemin S (2015). Maternal telomere length inheritance in the king penguin. Heredity.

[44] von Zglinicki T (2002). Oxidative stress shortens telomeres. Trends Biochem Sci.

[45] Houben JMJ, Moonen HJJ, van Shooten FJ, Hageman GJ (2008). Telomere length assessment: biomarker of chronic oxidative stress?. Free Radic Biol Medi.

[46] Zeichner SL, Palumbo P, Feng Y, Xiao X, Gee D, Sleasman J (1999). Rapid telomere shortening in children. Blood.

[47] Friedrich U, Schwab M, Griese EU, Fritz P, Koltz U (2001). Telomeres in neonates: new insights in fetal hematopoiesis. Pediatr Res.

[48] Hall ME, Nasir L, Daunt F, Gault EA, Croxall JP, Wanless S (2004). Telomere loss in relation to age and early environment in long-lived birds. Proc R Soc Lond.

[49] Price LH, Kao HT, Burgers DE, Carpenter LL, Tyrka AR (2013). Telomeres and early-life stress: an overview. Biol Psychi.

[50] Haussmann MF, Longenecker AS, Marchetto NM, Juliano SA, Bowden RM (2012). Embryonic exposure to corticosterone modifies the juvenile stress response, oxidative stress and telomere length. Proc R Soc Lond B.

[51] Tissier ML, Williams TD, Criscuolo F (2014). Maternal effects underlie ageing costs of growth in the Zebra Finch (*Taeniopygia guttata*). PlosOne.

[52] Remsen JV, Christie DA, Elliott A, del Hoyo J (2003). Family Furnariidae (ovenbirds). Handbook of the Birds of the World. Broadbills to Tapaculos.

[53] Moreno J, Merino S, Lobato E, Rodríguez-Girone MA, Vásquez RA (2007). Sexual dimorphism and parental roles in the thorn-tailed rayadito (furnariidae). Condor.

[54] Moreno J, Merino S, Vásquez RA, Armesto JJ (2005). Breeding biology of the thorn-tailed rayadito (furnariidae) in south-temperate rainforests of Chile. Condor.

[55] Quirici V, Venegas CI, González-Gómez PL, Castaño-Villa GJ, Wingfield JC, Vásquez RA (2014). Baseline corticosterone and stress response in the thorn-tailed rayadito (*aphrastura spinicauda*) along a latitudinal gradient. Gen Comp Endocrinol.

[56] Criscuolo F, Bertile F, Durant JM, Raclot T, Gabrielsen GW, Massemin S (2006). Body mass and clutch size may modulate prolactin and corticosterone levels in eiders. Physiol Biochem Zool.

[57] Blas J, Baos R, Anna C (2008). Stress in the nest: causes and consequences of adrenocortical secretion in developing birds. Recent advances in Non-mammalian adrenal gland research.

[58] Breuner CW, Orchinik M, Hahn TP, Meddle SL, Moore IT, Owen-Ashley NT (2003). Differential mechanisms for regulation of the stress response across latitudinal gradients. Am Physiol Regul Integr Comp Physiol.

[59] Fridolfsson AK, Ellegren DH (1999). A simple and universal method for molecular sexing of non-ratite birds. J Avian Biol.

[60] Cawthon RM (2002). Telomere measurement by quantitative PCR. Nucleic Acids Res.

[61] Criscuolo F, Bize P, Nasir L, Metcalfe NB, Foote CG, Griffiths K (2009). Real-time quantitative PCR assay for measurement of avian telomeres. J Avian Biol.

[62] Sokal RR, Rohlf FJ (1995). Biometry.

[63] Jakob EM, Marshall SD, Uetz G (1996). Estimating fitness: a comparation of body condition indices. Oikos.

[64] Green AJ (2001). Mass/length residuals: measures of body condition or generators of spurious results?. Ecology.

[65] Stier A, Viblanc VA, Massemin-Challet S, Handrich Y, Zahn S, Rojas ER (2014). Starting with a handicap: phenotypic differences between early- and late-born king penguin chicks and their survival correlates. Fun Ecol.

[66] Tarry-Adkins JL, Chen JH, Smith NS, Jones RH, Cherif H, Ozanne SE (2009). Poor maternal nutrition followed by accelerated postnatal growth leads to telomere shortening and increased markers of cell senescence in rat islets. The FASEB J.

[67] Schultner J, Moe B, Chastel O, Bech C, Kitaysky AS (2014). Migration and stress during reproduction govern telomere dynamics in a seabird. Biol Lett.

[68] Voillemot M, Hine K, Zahn S, Criscuolo F, Gustafsson L, Doligez B (2012). Effects of brood size manipulation and common origin on phenotype and telomere length in nestling collared flycatchers. BMC Ecol.

[69] Nettle D, Monaghan P, Gillespie R, Brilot B, Bedford T, Bateson M (2015). An experimental demonstration that early-life competitive disadvantage accelerates telomere loss. Proc R Soc Lond B.

[70] Love OP, Chin EH, Wynne‐Edwards KE, Williams TD (2005). Stress hormones: a link between maternal condition and Sex‐biased reproductive investment. Am Nat.

[71] Almasi B, Rettenbacher S, Muüeller C, Brill S, Wagner H, Jenni L (2012). Maternal corticosterone is transferred into the egg yolk. Gen Comp Endocrinol.

[72] Kitaysky AS, Wingfield JC, Piatt JF (1999). Dynamics of food availability, body condition and physiological stress response in breeding Black-legged Kittiwakes. Func Ecol.

[73] Harding AMA, Kitaysky AS, Hall ME, Welcker J, Karnovsky NJ, Talbot SL (2009). Flexibility in the parental effort of an arctic-breeding seabird. Func Ecol.

[74] González J, Wink M (2010). Genetic differentiation of the thorn-tailed rayadito *aphrastura spinicauda* (furnariidae: Passeriformes) revealed by ISSR profiles suggests multiple palaeorefugia and high recurrent gene flow. Ibis.

[75] Yáñez DI, Quirici V, Castaño-Villa GJ, Poulin E, Vásquez RA (2015). Isolation and characterisation of eight microsatellite markers of the thorn-tailed rayadito *aphrastura spinicauda*. Ardeola.

[76] van Rossum EFC, Lamberts SWJ (2004). Polymorphisms in the glucocorticoid receptor gene and their associations with metabolic parameters and body composition. Recent Prog Horm Res.

[77] Mondloch CJ (1995). Chick hunger and begging affect parental allocation of feedings in pigeons. Anim Behav.

[78] Sacchi R, Saino N, Galeotti P (2002). Features of begging calls reveal general condition and need of food of barn swallow (*Hirundo rustica*) nestlings. Behav Ecol.

[79] Hayward LS, Wingfield JC (2004). Maternal corticosterone is transferred to avian yolk and may alter offspring growth and adult phenotype. Gen Comp Endocrinol.

[80] Sheriff MJ, Krebs CJ, Boonstra R (2010). The ghosts of predators past: population cycles and the role of maternal effects under fluctuating predation risk. Ecology.

[81] Marra PP, Holberton RL (1998). Corticosterone levels as indicators of habitat quality: effects of habitat segregation in a migratory bird during the non-breeding season. Oecologia.

[82] Lanctot RB, Hatch SA, Gill VA, Eens M (2003). Are corticosterone levels a good indicator of food availability and reproductive performance in a kittiwake colony?. Horm Behav.

[83] Buck CL, O’Reilly KM, Kildaw SD (2007). Interannual variability of Black-legged Kittiwake productivity is reffected in baseline plasma corticosterone. Gen Comp Endocrinol.

[84] Kitaysky AS, Piatt JF, Wingfield JC (2007). Stress hormones link food availability and population processes in seabirds. Marine Ecol Prog Series.

[85] Martínez-Mota R, Valdespino C, Sánchez-Ramos MA, Serio-Silva JC (2007). Effects of forest fragmentation on the physiological stress response of black howler monkeys. Anim Conserv.

[86] Jenni-Eiermann S, Glaus E, Grüebler M, Schwabl H, Jenni L (2008). Glucocorticoid response to food availability in breeding barn swallows (*Hirundo rustica)*. Gen Comp Endocrinol.

[87] Blas J, Baos R, Bortolotti MTA, Hiraldo F (2005). A multi-tier approach to identifying environmental stress in altricial nestling birds. Func Ecol.

[88] Zanette L, Doyle P, Tremont SM (2000). Food shortage in small fragments: evidence from an area-sensitive passerine. Ecology.

[89] Dunn PO, Winkler DW, Fiedler W, Berthold P, Moller AP (2010). Effects of climate change on timing of breeding and reproductive success in birds. Effects of climate change on birds.

[90] Tallmon DA, Draheim HM, Mills LS, Allendorf FW (2002). Insights into recently fragmented vole populations from combined genetic and demographic data. Mol Ecol.

[91] Burke DM, Nol E (1998). Influence of food abundance, nestsite habitat, and forest fragmentation on breeding ovenbirds. Auk.

